# Human-to-mouse prion-like propagation of mutant huntingtin protein

**DOI:** 10.1007/s00401-016-1582-9

**Published:** 2016-05-24

**Authors:** Iksoo Jeon, Francesca Cicchetti, Giulia Cisbani, Suji Lee, Endan Li, Jiwoo Bae, Nayeon Lee, Ling Li, Wooseok Im, Manho Kim, Hyun Sook Kim, Seung-Hun Oh, Tae-Aug Kim, Jung Jae Ko, Benoit Aubé, Abid Oueslati, Yun Joong Kim, Jihwan Song

**Affiliations:** 1 CHA Stem Cell Institute, CHA University, Room 604, CHA Bio Complex, 335 Pangyo-ro, Bundang-gu, Seongnam-si, 13488 Gyeonggi-do Republic of Korea; 2 Centre de recherche du CHU de Québec (CHUQ), Québec, QC G1V 4G2 Canada; 3 Département de Psychiatrie and Neurosciences, Université Laval, Québec, QC G1V 0A6 Canada; 4 Department of Neurology, Seoul National University Hospital, Seoul, Korea; 5 Département de Médecine Moléculaire, Université Laval, Québec, QC G1V 0A6 Canada; 6 Ilsong Institute of Life Science, Hallym University, Anyang, Korea

**Keywords:** Huntington’s disease, Full-length mutant huntingtin, Human fibroblasts, Human-induced pluripotent stem cells, Exosomes

## Abstract

Huntington’s disease (HD) is an autosomal dominant neurodegenerative disorder of the central nervous system (CNS) that is defined by a CAG expansion in exon 1 of the huntingtin gene leading to the production of mutant huntingtin (mHtt). To date, the disease pathophysiology has been thought to be primarily driven by cell-autonomous mechanisms, but, here, we demonstrate that fibroblasts derived from HD patients carrying either 72, 143 and 180 CAG repeats as well as induced pluripotent stem cells (iPSCs) also characterized by 143 CAG repeats can transmit protein aggregates to genetically unrelated and healthy host tissue following implantation into the cerebral ventricles of neonatal mice in a non-cell-autonomous fashion. Transmitted mHtt aggregates gave rise to both motor and cognitive impairments, loss of striatal medium spiny neurons, increased inflammation and gliosis in associated brain regions, thereby recapitulating the behavioural and pathological phenotypes which characterizes HD. In addition, both in vitro work using co-cultures of mouse neural stem cells with 143 CAG fibroblasts and the SH-SY5Y human neuroblastoma cell line as well as in vivo experiments conducted in newborn wild-type mice suggest that exosomes can cargo mHtt between cells triggering the manifestation of HD-related behaviour and pathology. This is the first evidence of human-to-mouse prion-like propagation of mHtt in the mammalian brain; a finding which will help unravel the molecular bases of HD pathology as well as to lead to the development of a whole new range of therapies for neurodegenerative diseases of the CNS.

**Electronic supplementary material:**

The online version of this article (doi:10.1007/s00401-016-1582-9) contains supplementary material, which is available to authorized users.

## Introduction

Prominent neuronal loss typifies neurodegenerative disorders and the presence of abnormally structured or misfolded proteins has conferred to these conditions the title of proteinopathies. In recent years, a surge of publications has consistently reported experimental evidence for the spreading of such abnormal proteins, including β-amyloid, Tau, SOD1, TDP-43 and FUS [22, 44]. It was perhaps the more recent observation of Lewy body pathology in patients with Parkinson’s disease (PD) in receipt of fetal ventral mesencephalic transplants [23, 24] that propelled the theory that pathogenic species of proteins can spread in a prion-like fashion [4, 11, 22, 35, 36, 38, 41, 44]. Indeed, it was subsequently shown that α-synuclein can be released and taken up by neurons [13, 19, 23, 24] and thereby seed pathology both in vitro and in vivo. Intracerebral inoculation of brain homogenates derived from aged α-synuclein transgenic mice, or injection of synthetic α-synuclein-preformed fibrils, accelerates the formation of α-synuclein protein aggregates and precipitates neurological dysfunction in rodents [4, 15, 22, 26, 27, 35, 44]. In addition to the possibility of an intercellular trans-synaptic transport of proteins, α-synuclein has been shown to co-localize with markers of endosomes and exosomes, which could represent a route by which it is transferred [2, 4, 11, 16, 22, 35, 38, 41, 44].

However, in Huntington’s disease (HD), like in all trinucleotide disorders of the CNS, the classical dogma purports that the abnormal protein, in this case mutant huntingtin (mHtt), causes cellular dysfunction through a cell-autonomous manner that results in aggregation, inclusion body formation and cell death [8, 13, 19, 23, 24, 48]. Yet, despite the fact that HD is an autosomal dominant disorder, recent publications have challenged the assumption that the pathology emerges from a purely cell-autonomous process [3, 7, 37]. The severity of the clinical features—based on movement disorders, cognitive dysfunction and psychiatric problems [39]—in the presence of an expanded CAG repeat variably correlate with disease phenotype, which implies that additional extrinsic factors are responsible for the severity of symptoms and the timing of disease onset [18]. The recent observation of aggregates of the mHtt gene product within fetal striatal allografts in patients with HD provides strong evidence for the existence of non-cell-autonomous mechanisms of action for pathological protein spread in genetic disorders as well [7]; a theory which is gaining support both in vitro and in vivo [12, 13, 19, 26, 27, 30, 31].

Here, we addressed the contribution of non-cell-autonomous mechanisms of mHtt spread to disease onset and development by transplanting human fibroblasts, induced pluripotent stem cells (iPSCs) or exosomes derived from HD cases expressing various CAG repeat lengths into normal wild-type neonatal mice. The data collected provide compelling evidence that the intrinsic expression of the mutated gene is not needed for the HD-related phenotype nor pathology to develop.

## Materials and methods

### Establishment of fibroblast cell lines

Skin fibroblasts were isolated from a 6-year old male juvenile HD patient carrying 143 CAG repeats (HD143F). The procedures were approved by the Institutional Review Board of Seoul National University Hospital (Seoul, Korea). Two additional fibroblast lines were obtained from Coriell Cell Repositories (Camden, NJ, USA), originating from a 20-year old female HD patient carrying 72 CAG repeats (HD72F; Cat. No.: GM04281) or an additional 6-year old male juvenile HD patient carrying 180 CAG repeats (HD180F; Cat. No.: GM09197). As a control, embryonic fibroblasts (hEF) obtained from aborted human embryos were used following approval by the Institutional Review Board of CHA Gangnam Medical Center (Seoul, Korea). Fibroblasts were maintained in Dulbecco’s Modified Eagle’s Medium (DMEM) high glucose (Welgene, Korea), supplemented with 10 % fetal bovine serum (Invitrogen) and 1 % penicillin/streptomycin (Sigma-Aldrich) grown on 0.2 % gelatin-coated culture dish. For the transplantation of fibroblasts, they were dissociated using 0.25 % trypsin–EDTA and resuspended in DMEM high glucose with 30 µM Y-27632 (Tocris) following a centrifugation at 1200 rpm for 3 min.

### Genomic DNA isolation and PCR

Given that the focus of the manuscript is on the experiments performed with the 143 CAG fibroblasts, we will primarily describe these cells. Genomic DNA was, therefore, isolated from hEF and HD143F using genomic DNA isolation buffer (10 mM Tris–HCl, pH 8.0, 200 Mm NaCl, 10 mM EDTA, 0.5 % SDS, 100 µg/ml Proteinase K (Roche), incubated at 55 °C for 3 h, followed by phenol/chloroform extraction and ethanol precipitation. The purified genomic DNA was dissolved in TE buffer (10 mM Tris–HCl, pH 8.0, 1 mM EDTA) and used to amplify the *htt* gene. PCR amplification was performed in primeSTAR premix (Takara), Pfu HQ buffer (GeneAll) using primers designed to anneal 638 and 1010 bps for hEF and HD143F, respectively. The primer’s sequences were as follows: forward primer (Htt-UTR FW) 5′-ATTGGCAGAGTCCGCAGGCTAG-3′ and reverse primer (Htt intron 1-2 Rev) 5′-GCTGGGTCACTCTGTCTCTG-3′ (Supplemental Fig. 1a). Additional characterizations included the expression of mHtt using EM48, karyotypes and growth curves (Supplemental Fig. 1a).

### Preparation of iPSCs

HD143-iPSCs were generated using the episomal method [34]. Briefly, HD143F and hEF were electroporated with the episomal vectors using Neon™ Transfection System (Invitrogen), and the resulting iPSC colonies were maintained and expanded, which formed stable iPSC lines after passage 10 (P10). Established iPSC lines were examined for pluripotency using the RT-PCR analysis. They were immunostained with antibodies against pluripotency markers, such as Oct4, SOX2, Tra-1-81, and SSEA4 (data not shown) as well as for the expression of mHtt using EM48 (Supplemental Fig. 2). We further performed karyotype analysis, based on GTG-banding patterns (Supplemental Fig. 2). After the characterization of established iPSC lines, each iPSC line was differentiated into neural precursor cells (NPCs) according to previously published methodology [30] for implantation into the neonatal mouse brain.

### Injection of fibroblasts or iPSC-derived NPCs into the neonatal mouse brain

Adult CF-1 mice were purchased from the Jackson Laboratory and housed in a temperature and light-controlled environment (22 °C, a 12-h cycle) at the CHA University animal research center. Mouse pups were cryoanesthetized at post-natal day 2 (P2) after direct contact with ice for 3 min. The anesthetised pup’s head was gently held, and the ventricles were visualized by transillumination, keeping the head over a light source. A total of 100,000 fibroblasts (HD72F, HD143F, HD180F or hEF cells) or iPSCs (HD143-iPSC or epi-iPSC cells) resuspended in a 1 µl volume—after overnight incubation with 1 µg/ml bisbenzimide (Hoechst 33342, Sigma-Aldrich) at 37 °C—were injected into the lateral ventricles bilaterally using a 29G Hamilton syringe (Hamilton Company). The transplantation procedure was carried out quickly to minimize pups handling (approximately 5 min per pup) and, therefore, to reduce the risks of cannibalism when animals were returned to their mothers. All experiments were performed in accordance with the guidelines of the CHA University IACUC (Institutional Animal Care and Use Committee; IACUC090012).

### Behavioural tests

**Fig. 1:**
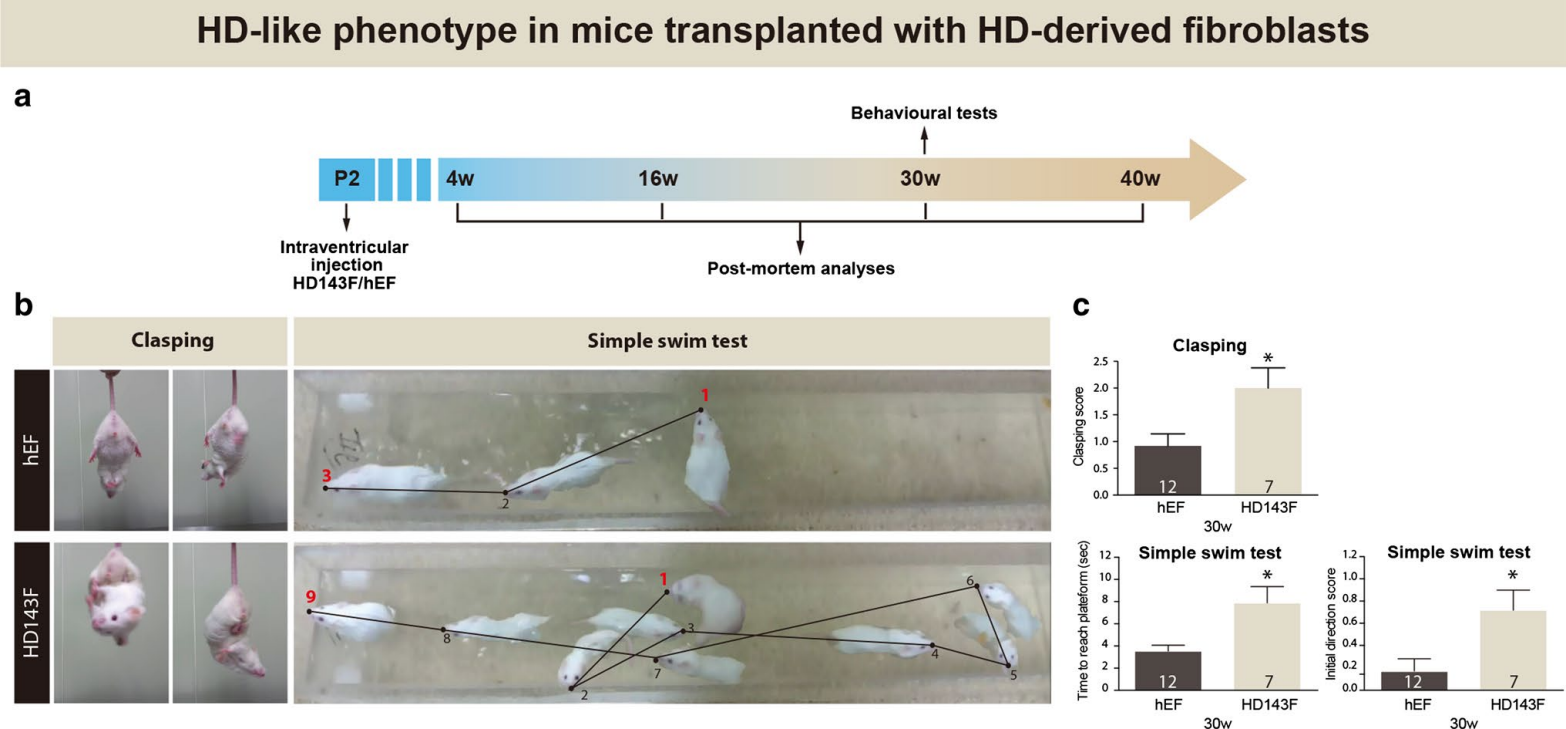
Development of HD-associated behavioural phenotypes following intraventricular injection of HD patient-derived fibroblasts. **a** Timeline of in vivo experimentation using fibroblasts derived from an HD patient carrying 143 CAG repeats. **b** Representative images of the clasping test as well as trajectories on the simple swim test. **c** Quantification of clasping reveals the manifestation of an HD phenotype in mice transplanted with fibroblasts collected from an HD patient (HD143F) compared to animals in receipt of control cells (hEF) at 30 weeks post-implantation. Plotting of the time to reach the platform and swimming direction scores (navigational memory) further reveals cognitive-related deficits in mice transplanted with fibroblasts positive for the HD gene at the same time point. Values are expressed as mean ± SEM. Statistical analyses were performed using the Student’s *t* test. *p < 0.05 compared to hEF. The number of mice used in each group is indicated in each *column* of the graphs. *HD143F* fibroblasts derived from an HD patient with 143 CAG repeats; *hEF* human embryonic fibroblasts; *P2* post-natal day 2; *w* weeks

Motor and cognitive deficits were assessed in mice that received either HD patient-derived fibroblasts or iPSCs. Clasping and simple swim test were evaluated in mice transplanted with HD143F at 30 weeks of age (Fig. 1a), while the simple swim test, forced swim test, rotarod and grip strength tests were performed in mice that received HD72F, HD180F and HD143-iPSCs. In these experiments, the behavioural measures were taken at 34, 36 and 38 weeks of age (Supplemental Fig. 3 a, d).

#### Clasping test

The clasping test, a classic measure of the HD phenotype [10], was performed by suspending the mice by the tail 10 cm above the cage for 1 min. Mice demonstrating HD-like features clasped both front and hind legs and twisted their body [25]. Animals were scored using a scale that ranged from 0 (the hindlimbs consistently splayed outward) to 3 (both hindlimbs are entirely retracted and touching the abdomen for more than 50 % of the time suspended).

#### Simple swim test

The simple swim test was chosen as a measure of cognitive impairments and procedural learning, as previously performed in HD animals [40, 46]. Mice were placed in the center of a linear swimming chamber (76 × 13 cm; water depth, 9 cm; platform, 6 × 13 cm) facing away from the escape platform. The amount of time required to reach the platform and the initial swimming direction was recorded for each trial. Swimming towards the platform was arbitrarily given a score of 0, whereas swimming away from the platform was given a score of 1. Mice were trained for 3 days with three pairs of two consecutive trials spaced 2 h apart. For each mouse, the average of the three trials on the last day of testing was used for the analysis [40].

#### Forced swim test

The forced swim test is commonly used to identify depression-like behaviour but also motility in HD mice [33, 40]. This test was conducted by placing the mice in a glass cylinder (25-cm tall × 19-cm wide) filled with warmed water (23–25 °C) to a depth of 15 cm for a period of 6 min. The test sessions were recorded by a video camera. The last 4 min of the test session was scored using a time-sampling technique to rate the predominant behaviour over 1 s interval. A number of parameters were measured (swimming, immobility and climbing).

#### Rotarod test

We used an accelerating rotarod protocol (San Diego Instruments) to test motor coordination and gait changes, as routinely performed in the characterization of an HD phenotype in mice [10]. Accelerations ranged from 0 to 45 rpm over a period of 2 min. Mice were trained for 3 days, two trials per day. Following the training period, mice were tested for three consecutive trials in a single day and allowed 1.5 h rest time between trials. The rotarod was wiped clean with ethanol between each subject and trial.

#### Grip strength test

Grip strength was finally used as an additional assessment of motor function [29]. The apparatus (San Diego Instruments) is comprised of an adjustable grip (6 cm wide, 0°–45°) connected to the digital gauge. For this measure, the mouse was lifted by the tail, so that its forepaws could grasp the grip. The mouse was then gently pulled backward by the tail until the wire was released. The maximal force exerted before the mouse lost its grip was recorded. Each mouse was tested for nine trials, and the average of three highest scores was used for subsequent analysis.

### Post-mortem analyses

#### Tissue processing

Mice were sacrificed at 4, 16, 30 and 40 weeks following the injection of HD143F (Fig. 1a) or 40 weeks following the implantation of HD72F, HD180F or HD143-iPSCs (Supplemental Fig. 3a, d**)**. They were subjected to intracardiac perfusion with phosphate-buffered saline (PBS) and 4 % paraformaldehyde (PFA) under deep anesthesia with 1 % ketamine (30 mg/kg) and xylazine hydrochloride (4 mg/kg). Brains were collected, post-fixed in 4 % PFA overnight and subsequently stored in 30 % sucrose for cryoprotection. Coronal 40 µm-thick brain sections were obtained using a sliding microtome (Leica), serially collected in anti-freeze solutions and kept at −20 °C until use for immunohistochemistry/immunofluorescence. In this study, only the post-mortem evaluation related to the HD143F experiment is presented.

#### Immunofluorescent staining

Free-floating sections were washed in PBS (2 × 15 min) followed by 1 min incubation in PBS containing 1 % sodium dodecyl sulfate (SDS). After three additional washes in PBS with 0.3 % triton X-100, sections were blocked in 5 % normal horse (Vector Laboratories) or 10 % donkey serum (Sigma-Aldrich) followed by incubation overnight with the primary antibody for either CR (1:100, Cat. No. 769914, Swant), DARPP-32 (1:200, Cat. No. #2306, clone 19A3, Cell Signaling), ED1 (1:100, Cat. No. MAB1435, Millipore), EM48 (1:50, Cat. No. MAB5374, Millipore), GFAP (1:100, Cat. No. G5601, Promega), Iba1 (1:100, Cat. No. 019-19741 Wako), MAP2 (either 1:200, Cat. No. AB5622, Millipore or 1:100, Cat. No. 17490-1-AP, Protein Tech), hMito (1:200, Cat No. MAB1273, clone 113-1, Millipore), MW7 (1:100, obtained from the Developmental Studies Hybridoma Bank), NeuN (1:500, Cat. No, MAB377, clone A60, Millipore) or Ubiquitin (1:1000; Cat. No. Z0458, Dako). Following a series of washes in PBS, sections were incubated with the appropriate fluorescently-conjugated antibodies for 2 h at room temperature. Finally, following three additional washes, sections were incubated in PBS containing 1 µg/ml DAPI (Roche), washed and coverslipped with Vectashield (Vector Laboratories).

Note that for the MAP2-MW7 double immunofluorescent staining, sections were further incubated in 4 % PFA pH 7.4 for 1 h, followed by three washes in KPBS, and blocked in 1 % BSA, 0.4 % Triton X-100 and 5 % NGS diluted in KPBS for 30 min. Incubation in the primary antibody exceeded 48 h at 4 °C. After three additional washes in KPBS, the sections were incubated in the appropriate secondary antibody diluted in a blocking solution and counterstained with DAPI 0.022 %.

#### Confocal laser-scanning microscopy

Confocal laser scanning microscopy was performed using LSM510 (Carl Zeiss) and Olympus FV500 (Olympus America Inc.) confocal laser-scanning microscopes. Images were acquired by sequential scanning and optimized by a two-frame Kalman filter and analyzed using the Fluoview SV500 imaging software 4.3 (Olympus America Inc., Melville, USA).

#### Immunohistochemistry

A similar protocol as described above was used for the immunohistochemical staining of DARPP-32 (1:200). Following incubation with the primary antibody, sections were incubated with biotinylated anti-rabbit secondary antibody (1:500, Vector Laboratories) and the avidin–biotin–peroxidase complex (Vector Laboratories) according to manufacturer’s instructions. Sections were developed in 0.05 % 3-3′-diaminobenzidine tetrahydrochloride (DAB) and 0.003 % hydrogen peroxide in 0.1 M Tris–Cl (pH 7.5). Following the DAB reaction, sections were mounted on glass slides. All sections were finally air-dried, dehydrated in ascending grades of ethanol, cleaned in xylene, and coverslipped with DPX mounting media (EM Science). Images were acquired with a phase-contrast microscope (Eclipse E600, Nikon).

#### Quantification of DARPP-32, GFAP and ED1 immunostaining

The counts of DARPP-32^+^, GFAP^+^ or ED1^+^ cells were performed by sampling nine different areas (1 mm × 1 mm each) of the striatum from three different animals per group at 4, 16, 30 and 40 weeks (for DARPP-32) or 30 and 40 weeks (for GFAP and ED1) post-injection using the ImageJ software (Image J, NIH).

#### Quantification of EM48 immunofluorescent staining intensity

Immunoflurorescent staining intensity for EM48-positive signals was measured from three different regions of striatum or cortex in the mouse brains at 40 weeks following implantation with HD143F or HD143-iPSC using the ImageJ software, on maximum projection images resulting from the superimposition of the single z-stacks, as described previously [28].

### Co-culture of mouse neural stem cells with fibroblasts

Neural stem cells (NSCs) were isolated from the cortical region of developing mouse (*FVB/NJ*) at embryonic day 12.5 and were maintained in NSC medium containing DMEM/F12 (Gibco), N2 supplement (1X, Gibco), bFGF (10 ng/ml, Peprotech), EGF (10 ng/ml, Millipore) and antibiotic/antimycotic solution (1X, Welgene, Korea) on tissue culture dishes pre-coated with poly-l-ornithine (PLO; 15 µg/ml, Sigma-Aldrich) and laminin (5 µg/ml, Sigma-Aldrich). To induce neural differentiation, NSCs were detached using TrypLE select (1X, Gibco), and the dissociated cells (1 × 10^6^ cells) were transferred to 60 mm^2^ bacterial *Petri* dishes (SPL, Korea) for 2 days to induce the formation of neurospheres (NSs). Two-day old NSs were transferred to four-well plates (Nunc) containing pre-plated hEF or HD143F cells with the initial seeding density of 5 × 10^4^ cells per well (approximately 15–20 NSs in each well). Medium was changed every other day. Transferred NSCs were further differentiated for one week in the medium containing DMEM/F12 (Gibco), 0.1 mM NEAA (Gibco), 0.1 mM β-mercaptoethanol (Invitrogen), 0.2 mM ascorbic acid (Sigma-Aldrich), N2 supplement (Gibco) and BDNF (10 ng/ml, R&D Systems). After 1 week, differentiated cells were fixed using 4 % paraformaldehyde for 15 min, followed by washes with PBS containing 0.1 % Triton X-100 for three times. Immunocytochemistry was performed using antibodies against MAP2 (1:200, Millipore) and EM48 (1:100, Millipore).

### Exosome extraction and characterization

Exosomes were isolated by ExoQuick-TC™ Exosome Precipitation Solution (System Biosciences) from hEF and HD143F following the manufacturer’s protocol. Briefly, supernatants of media containing exosome-free fetal bovine serum was collected from fibroblasts after 24 h in culture and mixed with ExoQuick-TC exosome Precipitation Solution. After refrigeration overnight, the mix was centrifuged at 1500×*g* for 30 min. Supernatant was removed, and the pellet was resuspended in lysis buffer for western blot analysis.

### Western blot analysis

Cells and exosomes were lysed in ice-cold cell lysis buffer containing 50 mM Tris/HCl (PH7.5), 2 mM EDTA, 150 mM NaCl, 1 % (v/v) Triton X-100, complete™ proteinase and phosphatase inhibitors (Roche), as described previously [5]. Lysates were loaded and subjected to SDS–polyacrylamide (4–20 %) electrophoresis and electroblotted onto PVDF membranes. After blocking in 5 % non-fat dry milk, membranes were incubated with mouse anti-polyglutamine (1C2, Cat. No. MAB1574, Millipore), mouse anti-total huntingtin (4C8, Cat. No. MAB2166, Millipore) and mouse anti-mutant huntingtin (EM48; 1:1000) antibodies. Exosomes were identified with the antibodies raised against CD9 (1:1000, Cat. No. EXOAB-CD9A-1, Systems Biosciences), CD63 (1:1000, Cat. No. EXOAB-CD63A-1, Systems Biosciences), CD81 (1:1000, Cat. No. EXOAB-CD81A-1, Systems Biosciences) and HSP70 (1:1000, Cat. No. EXOAB-Hsp70A-1, Systems Biosciences). Membranes were incubated with the appropriate secondary antibody (goat anti-mouse or anti-rabbit) according to the manufacturer’s instructions, followed by the addition of the chemiluminescent detection reagent (Millipore). Bands were visualized using a ChemiDoc™ XRS + system (Bio-Rad) and quantified using the image lab software (Bio-Rad).

### Cell line and culture conditions

The SH-SY5Y (human neuroblastoma) cell line was cultured in DMEM (Sigma-Aldrich) supplemented with 10 % heat inactivated FBS (Sigma-Aldrich), 2 mM l-glutamine (Invitrogen) and 1X penicillin/streptomycin solution (Sigma-Aldrich). Cells were plated in 10 cm^2^ petri dishes and transfected using FastFect (Feldan) according to the manufacturer’s instructions. Three different constructs were introduced into the cells. cDNA coding for human huntingtin Exon 1, harboring different CAG repeats (Q19 or Q103) fused to GFP as well as cDNA coding for GFP alone were cloned into the pAAV-CMV-MCS backbone (Stratagene). All constructs were verified by sequencing. Twenty hours post-transfection, the culture medium was replaced with DMEM without FBS for an additional 24 h after which cells, and media were collected. Cells were resuspended in lysis buffer containing phosphatase and protease inhibitors (Thermo Scientific). Part of the collected medium was used to perform an LDH assay (Life technologies) according to the manufacturer’s instructions, while the remaining medium was used for exosome extraction.

### Exosome extraction from SH-SY5Y cells conditioned media

The media were centrifuged for 30 min at 10,000×*g* at 4 **°**C to eliminate cell debris and then concentrated using Amicon Ultra 4 ml centrifugal filters prior to exosome isolation, as previously described [45]. Briefly, the concentrated medium underwent a series of ultracentrifugation. After a first centrifugation at 20,000×*g* for 1 h and 30 min at 4 **°**C, the medium was collected and centrifuged again at 100,000×*g* for 1 h at 4 **°**C. The pellet containing exosomes were finally resuspended in phosphate buffer. The presence of exosomes was validated with a Zetasizer Nano series ZS (Malvern) using the software Zetasizer 7.02 [32].

### Western blot analysis from SH-SY5Y cells

Laemmli buffer was added to the cell extract, to the concentrated medium as well as exosome preparation and ultimately heated at 95 **°**C for 15 min. Samples were loaded and subjected to SDS-polyacrylamide (10 %) gel electrophoresis. Proteins were immunoblotted onto 0.45 µm Immobilon PVDF membranes (Millipore) and blocked in 5 % non-fat dry milk and 1 % BSA in PBS-tween. Membranes were incubated with mouse anti-polyglutamine (1C2; 1:1000), rabbit anti CD-63 (1:1000; Cat No. ab199921, Abcam) and anti β-actin (1:10,000; Cat No. G043, ABM Inc.) using appropriate secondary antibodies, such as goat anti-rabbit or anti-mouse (1:25,000; Jackson Immunoresearch), followed by the addition of the chemiluminescence reagents (Luminata; Millipore). Band intensities were quantified using a myECL imager (Thermo Scientific).

### Incubation of SH-SY5Y cells in conditioned media and detection of incorporated mHtt

HEK cells transiently overexpressing GFP, GFP-mHtt-Q19 or GFP-mHtt-Q103 were cultured in DMEM (Sigma-Aldrich) supplemented with 10 % heat inactivated FBS (Sigma-Aldrich), 2 mM l-glutamine (Invitrogen) and penicillin/streptomycin (Sigma-Aldrich). Forty-eight hours post-transfection, the conditioned media were collected and centrifuged at 10,000×*g* for 10 min to remove cell debris. The supernatant was added to SH-SY5Y neuroblastoma and incubated for 5 days. The conditioned media were added three times over 5 days. After incubation, SH-SY5Y cells were treated with trypsin for 5 min to remove extracellular proteins and were collected and lysed in 1X lysis buffer. The total protein fraction was separated in SDS-polyacrylamide (10 %) gel and incorporated mHtt from the conditioned media that were detected by western blot using anti-mHtt (EM48) and anti-GFP antibodies.

### Treatment of NSCs with exosomes

NSCs, prepared as described above, were cultured in ultra-low attachment tissue culture dishes. One day later, neurospheres were transferred onto 12 mm glass coverslip pre-coated with poly-l-ornithine (PLO; 15 µg/ml, Sigma-Aldrich) and laminin (5 µg/ml, Sigma-Aldrich) in NSC medium. Following the attachment of the neurospheres, the culture medium was replaced with a medium containing DMEM F12, 1 % NEAA, 10 mM β-mercaptoethanol and 1 % antibiotic antimycotic solution supplemented with 1X N2, 2 mM l-glutamine, 3 mM d-glucose, 0.2 mM ascorbic acid and 10 ng/ml BDNF (NS medium) and 250 µg/ml of exosomes derived from HD143F and hEF (see timeline Fig. 5e). The medium was changed every other day. At day 4, the cells were fixed and immunostained for MAP2 (1:200) and anti-EM48 (1:100).

### Injection of exosomes into the neonatal mouse brain

The in vivo experiments pertaining to the injections of exosomes derived from the HD patient carrying 143 CAG repeats or from human embryonic fibroblast (hEF) (control) were performed using identical procedures as described above (see Sect. “Injection of fibroblasts or iPSC-derived NPCs into the neonatal mouse brain”). Exosome suspensions were injected into both ventricles (5 µg/each ventricle; a total of 10 µg per animal) using a 31G Hamilton syringe.

### Image preparation and statistical analyses

All images were prepared for illustration using Adobe Photoshop CS5 and Adobe Illustrator CS5. Data were analyzed with the one-factor analysis of variance (ANOVA) followed by a Tukey post hoc test or Student’s *t* tests, using the Statistical Analysis System (Enterprise 4.1, SAS Korea, Seoul Korea). Significance was accepted at the 95 % probability level. Data are presented as mean ± SEM.

## Results

### Injection of HD- patient-derived fibroblasts or iPSCs leads to HD-related motor and cognitive impairments in wild-type mice

We first evaluated the potential of HD patient-derived fibroblasts carrying the entire 170 kb genomic locus of the human *Htt* gene to generate an HD-associated phenotype in wild-type mice. Pathological fibroblasts displayed an amplification of exon 1 with 143 CAG repeats (HD143F), while controls (hEF) carried 19 CAG repeats (Supplemental Fig. 1a). The presence of the mutation was confirmed by PCR (Supplemental Fig. 1a), western blot analyses with an antibody directed against the polyQ expansion on exon 1 of the gene (Supplemental Fig. 1b) and immunoflurorescent staining using the mHtt-specific antibody EM48 (Supplemental Fig. 1c). Both HD143F and hEF showed normal karyotypes (Supplemental Fig. 1d), although HD143F was shown to grow slowly, compared with hEF (Supplemental Fig. 1e).

Upon phenotypic confirmation, HD143F or hEF cells were injected bilaterally into the lateral ventricles of wild-type pups at post-natal day 2 (P2), followed by behavioural measures (Fig. 1a). At 30 weeks, animals transplanted with HD143F cells displayed a measurable abnormal clasping response characteristic of HD mice [14] (Fig. 1b, c; *p* < 0.05 (see also Supplemental Video 1)). In the simple swim test—a measure of cognitive impairments and procedural learning—animals transplanted with HD143F required twice as much time to reach the visible escape platform than controls. They also displayed aberrant swimming direction patterns, reminiscent of impairments in both motor coordination and navigational memory (Fig. 1b, c; *p* < 0.05 (see also Supplemental Video 2)).

These results were corroborated using additional clones from HD patients carrying various CAG repeat lengths (Supplemental Fig. 3a–c). Using an identical experimental approach, newborn wild-type mice injected with fibroblasts derived from HD patients with either 72 or 180 CAG repeats (HD72F or HD180F) developed an HD behavioural phenotype over time (Supplemental Fig. 3a–c). At 34 weeks post-injection and onwards, animals began to show impairments on the simple swim test (time to reach the platform), the forced swim test (immobility time) (Supplemental Fig. 3b, c**)**, the grip strength (Supplemental Fig. 3c), and the rotarod (latency to fall) (Supplemental Fig. 3c). Aside from the forced swim test, which revealed greater impairments in mice treated with the HD180F (*p* < 0.05), there was no significant difference between the severity of deficits created with either cell type. Sham animals that underwent the same surgical procedures—omitting the injection of the cells or a corresponding vehicle—did not present with any behavioural impairments (Supplemental Fig. 3c).

These observations were further supplemented using an additional cell type, i.e., induced pluripotent stem cells derived from the same HD patient with 143 CAG repeats (HD143-iPSC) or controls (epi-iPSC) which were injected bilaterally into the lateral ventricles of wild-type pups at P2 (Supplemental Fig. 3d). At 36 and 38 weeks, animals transplanted with HD143-iPSC cells displayed measurable behavioural deficits characteristic of HD mice (Supplemental Fig. 3e, f; *p* < 0.05). In the simple swim test, animals transplanted with HD143-iPSC cells required significantly more time to reach the escape platform (see also Supplemental Video 3). They further displayed aberrant swimming direction patterns (Supplemental Fig. 3e, f; *p* < 0.05; see also Supplemental Video 3), impaired mobility, as revealed by the forced swim test (Fig. 3e, f; *p* < 0.05; see also Supplemental Video 4) as well as impairments in the grip strength test when compared to their control counterparts (Supplemental Fig. 3f; *p* < 0.05), although there were no significant differences between groups using the rotarod test (Supplemental Fig. 3f). Taken together, detectable abnormalities on these measures indicate the development of HD-like cognitive and motor-related features in mice transplanted with various cells carrying different clones of the mutated gene.

### Progressive striatal neuronal loss following intraventricular injection of HD patient-derived fibroblasts

**Fig. 2:**
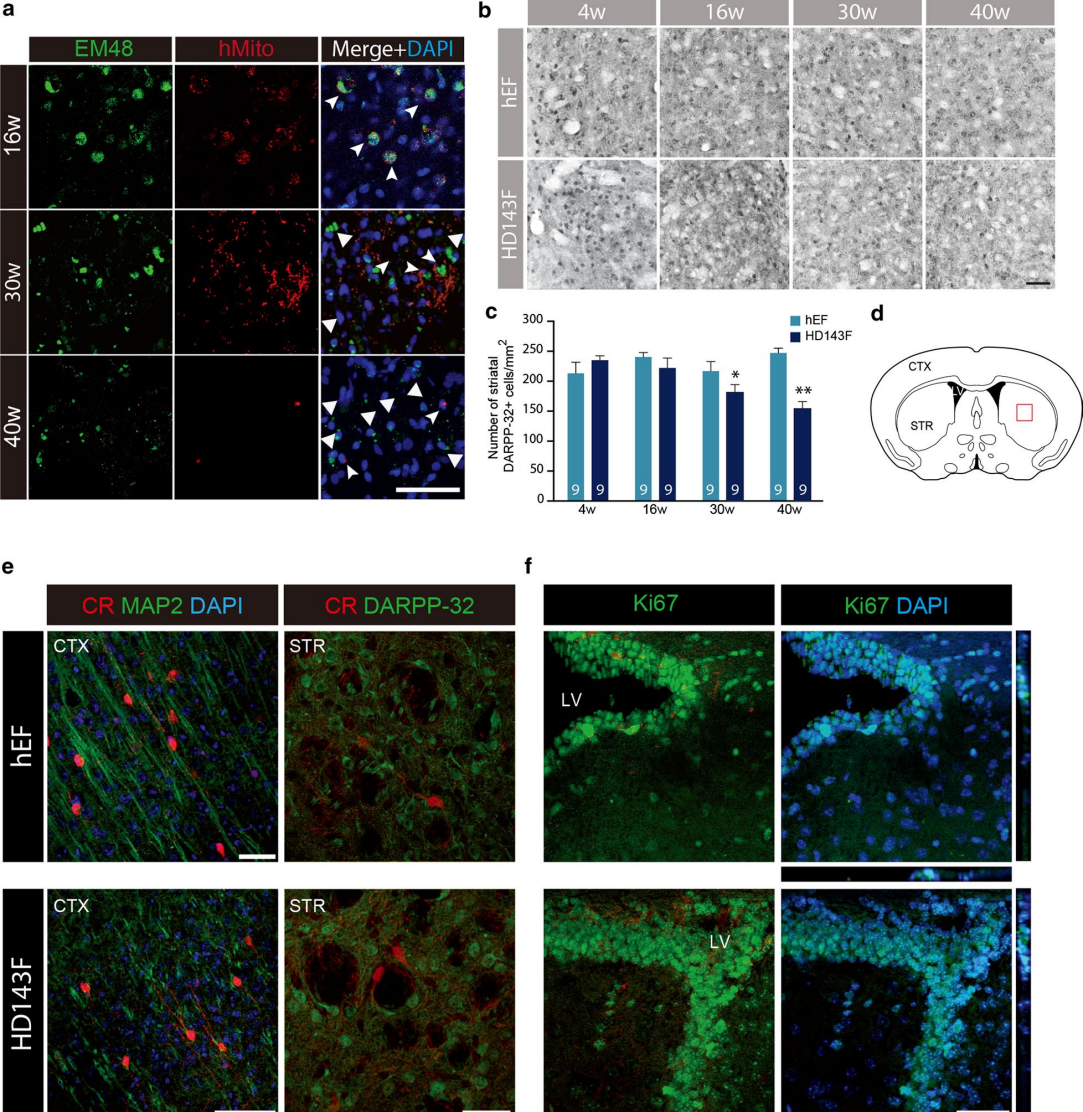
Progressive and targeted neuronal loss following intraventricular injection of HD patient-derived fibroblasts. **a** Immunoflurorescent staining of mHtt (EM48; *green*) and human mitochondria (hMito; *red*) demonstrating the expression of mHtt within the implanted HD143F cells at earlier time points (16 and 30 weeks post-injection; *indented arrowheads*) and the expression of mHtt aggregates alone (no longer associated with the hMito marker; *larger arrowheads*) at the later time points evaluated (30 and 40 weeks post-implantation). **b** Representative images of the immunohistochemical staining for DARPP-32 revealed with the chromogen diaminobenzidine (DAB) in the striatum of mice that received either hEF or HD143F cells. **c** Quantification of the progressive DARPP-32 striatal neuronal loss in mice that received HD143F or hEF at different time points. Values are expressed as mean ± SEM. *p < 0.05; **p < 0.005 compared to hEF group. The number of mice quantified in each group is indicated in each *column* of the graphs. **d** The atlas section of the mouse brain illustrates the area where images depicted in **a** and **b** were acquired (*red square*). **e** Sections were further stained for CR (*red*), present in subpopulations of interneurons, in combination with MAP2 (*green*) or DARPP-32 (*green*) and confirmed that, as in HD, interneurons are spared from pathology. This was exemplified in both the cortex (**e**, *left panels*) and in the striatum (**e**, *right panels*). **f** Finally, sections were stained for the presence of proliferating cells using Ki67 (*green*) and DAPI (*blue*) in the vicinity of the injection site (lateral ventricle) and confirmed the absence of tumour formation following the implantation of HD patient-derived fibroblasts. *Scale bars* **a** = 50 µm; **b** = 25 µm; **e** = 50 µm; **f** = 30 µm. *CR* calretinin; *DAPI* 4′,6-diamidino-2-phenylindole; *DARPP-32* dopamine- and cAMP-regulated phosphoprotein, Mr 32 kDa; *HD143F* fibroblasts derived from an HD patient expressing 143 CAG repeats; *hEF* human embryonic fibroblasts; *hMito* human mitochondria; *LV* lateral ventricle; *MAP2* microtubule associated protein 2; *w* weeks

To evaluate whether the observed motor and cognitive disturbances reflected an underlying neuropathological phenotype, we assessed the presence of mHtt focusing on one of the cell-type implanted, the HD patient-derived fibroblasts, using immunoflurorescent stainings for EM48 and hMito, human mitochondria (hMito), at 4 (data not shown), 16, 30 and 40 weeks post-injection (Fig. 2a). At 16 weeks post-transplantation, EM48 staining was closely associated with that of hMito but as time elapsed, hMito levels diminished and almost entirely vanished. EM48^+^ mHtt aggregates were scarcely found within the transplanted human fibroblasts but were detected mainly within the host parenchyma (Fig. 2a, 40w), indicating the transfer of the mHtt gene product irrespective of the survival of the donor cells.

We then investigated whether grafted human fibroblasts had an impact on the neighbouring striatum, in particular, the striatal medium spiny neurons, a neuronal population severely affected in HD. Consistent with the behavioural data (Fig. 1b), cell loss was apparent at 30 weeks following the transplantation of HD patient-derived fibroblasts and further progressed to 40 weeks post-transplantation, the ultimate time point we analyzed (Fig. 2b, c; *p* < 0.05, *p* < 0.005). In mice receiving control human fibroblasts, no changes in the number of DARPP-32^+^ neurons were observable at any of the time points analyzed (Fig. 2c). To assess the specificity of cell loss, we further stained brain sections for calretinin (CR), present in subpopulations of interneurons which are spared in HD [6]. As observed in HD pathology, CR-positive neurons, either cortical or striatal (Fig. 2e), did not show morphological changes nor compromised survival. Finally, to ensure that the loss of medium spiny neurons was not due to the proliferation of fibroblasts, sections were further stained for Ki67, a marker of cell proliferation, which demonstrated the presence of Ki67-positive cells was restricted to the ventricles, the site of injection. None of our observations revealed the presence of tumour formation (Fig. 2f).

### Evidence of mHtt transfer from HD patient-derived fibroblasts to host striatal cells

**Fig. 3:**
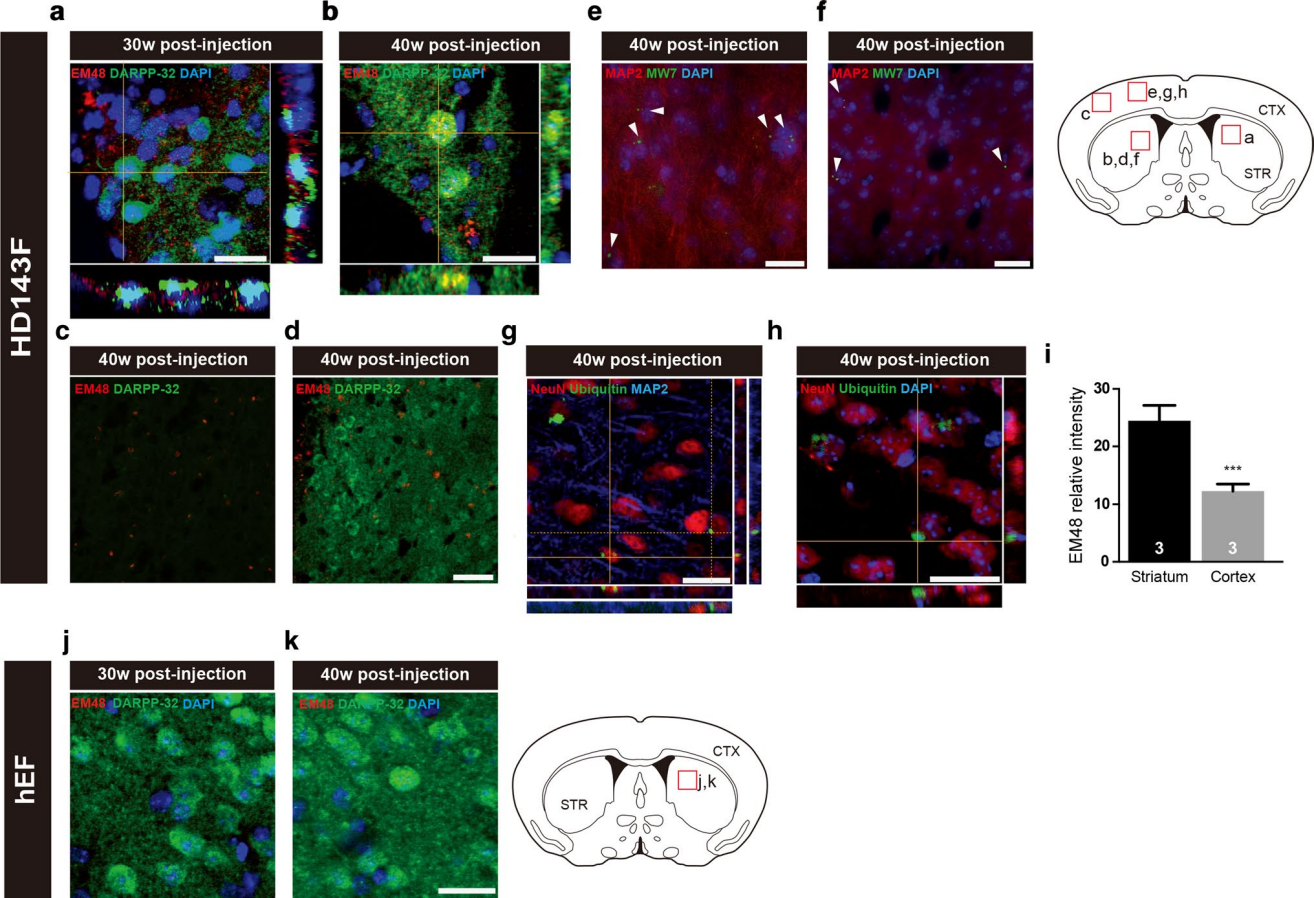
Evidence of cell-to-cell propagation of mHtt. Immunoflurorescent staining for mHtt (EM48; *red*), MW7 (*green*) or ubiquitin (*green*) combined with either DARPP-32 (*green*), MAP2 (*red*) or NeuN (*red*) reveals the propagation of mHtt from implanted HD patient-derived fibroblasts into both host striatal (**a**, **b**, **d**, **f**) and cortical cells (**c**, **e**, **g**, **h**) at 30 and 40 weeks post-transplantation, as assessed by confocal microscopy. The atlas section of the murine brain illustrates areas where images were acquired (*red squares*). Quantification of the relative intensity of the immnofluorescent EM48 staining further revealed that mHt aggregates were more abundant in the striatum than in the cortex (*i*). Values are expressed as mean ± SEM. Statistical analyses were performed using the Student’s *t* test. ***p < 0.001 compared to STR. The number of mice quantified in each group is indicated in each *column* of the graphs. Triple immunofluorescence for EM48, DARPP-32 and DAPI in mice injected with hEF showed, as expected, the absence of mHtt aggregates (**j**, **k**). *Scale bars* **a**, **b** = 20 µm; **c**, **d** = 50 µm; **e** = 10 µm; **f** = 20 µm; **g**, **h** = 25 µm; **j**, **k** = 20 µm). *CTX* cortex; *DAPI* 4′,6-diamidino-2-phenylindole; *DARPP-32* dopamine- and cAMP-regulated phosphoprotein, Mr 32 kDa; *HD143F* fibroblasts derived from an HD patient expressing 143 CAG repeats; *hEF* human embryonic fibroblasts; *NeuN* neuronal nuclei; *STR* striatum; *w* weeks

At 30 and 40 weeks post-transplantation, mHtt could be found within the host striatal neurons. Using triple immunofluorescent staining and confocal microscopy, EM48^**+**^ aggregates were detected within DARPP-32^+^ cellular elements, suggesting that mHtt had effectively been transferred from grafted cells of human origin to the host striatum at 30 (Fig. 3a) and 40 weeks post-injection (Fig. 3b, d) but also to the host cortex (Fig. 3c). We supplemented these observations using anti-MW7 and anti-ubiquitin antibodies, which confirmed that mHtt aggregates were indeed found within the host neurons, as demonstrated with both MAP2 and NeuN markers that are not expressed by human fibroblasts (Fig. 3e–h). A quantification of the relative immunofluorescent staining intensity for EM48 also revealed that mHtt aggregates were more abundant in the striatum than in the cortex (Fig. 3i). To validate the specificity of these findings, double immunofluorescent staining was also performed for DARPP-32 and EM48 in mice implanted with hEF control cells which remained devoid of mHtt staining (Fig. 3j, k).

### Implanted HD fibroblasts lead to a progressive gliosis and inflammation within the host brain

**Fig. 4:**
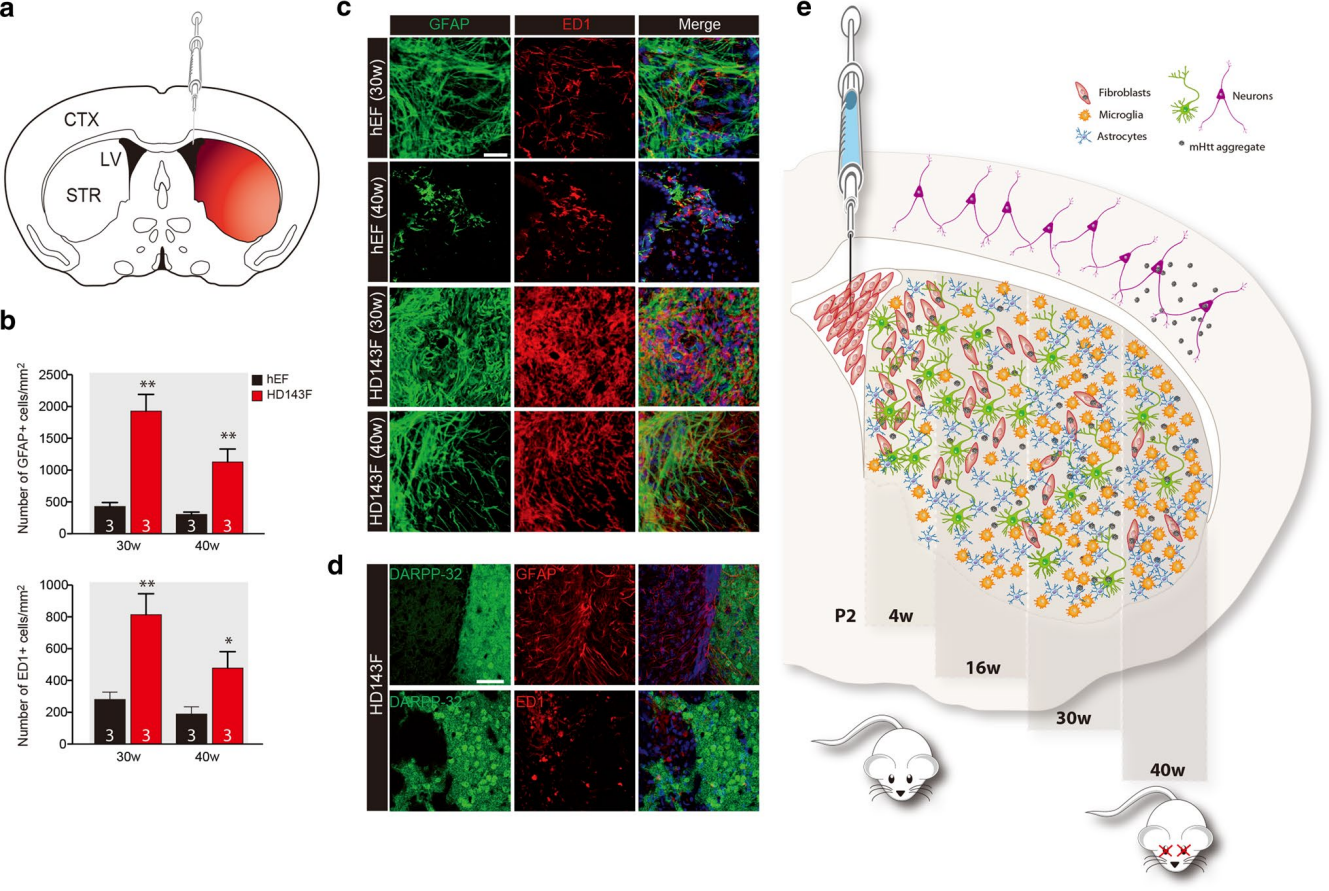
Development of an immune/inflammatory response following striatal neuronal loss in mice transplanted with HD patient-derived fibroblasts. **a** Schematic representation of the immune/inflammatory response gradient within the striatum illustrating that the strongest reaction is in the vicinity of the injection site. **b** The *bar graph* shows increased astroglial and microglial responses in the striatum of mice implanted with HD143F compared to those implanted with hEF at 30 and 40 weeks post-injection (*n* = 3 mice per group). **c** Representative images of astroglial (GFAP; *green*) and microglial (ED1; *red*) responses 30 and 40 weeks following the engraftment of fibroblasts from HD143F or hEF cells. Values are expressed as mean ± SEM. Statistical analyses were performed using the Student’s *t* test. *p < 0.05; **p < 0.005 compared to hEF group. The number of mice quantified in each group is indicated in each *column* of the graphs. **d** Representative images of the astroglial (GFAP; *red*) and microglial responses (ED1; *red*) surrounding neuronal (DARPP-32; *green*) and mHtt (EM48; *red*) staining. **e** Schematic summarizing the post-mortem observations following HD143F transplantation with respect to the corresponding timeline of analysis. *Scale bars* **b** = 30 µm; **d** = 50 µm. *CTX* cortex; *DARPP-32* dopamine- and cAMP-regulated phosphoprotein, Mr 32 kDa; *GFAP* glial fibrillar acidic protein; *HD143F* fibroblasts derived from an HD patient with 143 CAG repeats; *hEF* human embryonic fibroblasts; *LV* lateral ventricle; *STR* striatum

In addition to the loss of DARPP-32^+^ medium spiny neurons and the formation of EM48^+^ mHtt, increased gliosis and inflammation are evident in HD. Similarly, we observed that the significant striatal neuronal loss provoked by the injection of HD143F cells was accompanied by a significant increased gliosis in the vicinity of the lateral ventricles, the site of injection, with a gradient of activated glial cells that changed as a function of distance from transplant site (Fig. 4a). Indeed, a significant augmentation in the number of astrocytic (GFAP^+^) and microglial (ED1^+^) cells was quantified at both 30 and 40 weeks post-transplantation (Fig. 4b, c; *p* < 0.05, *p* < 0.005) and these cells were seen to populate the space originally occupied by the striatal tissue that had now been lost (Fig. 4d). Taken together, these findings show that human-derived mHtt aggregates can spread in the newborn mouse brain and translate into HD-related behavioural impairments, neuronal loss and gliosis through adulthood (Fig. 4e).

### Impact of secreted mHtt on cell morphology and neuronal differentiation

**Fig. 5:**
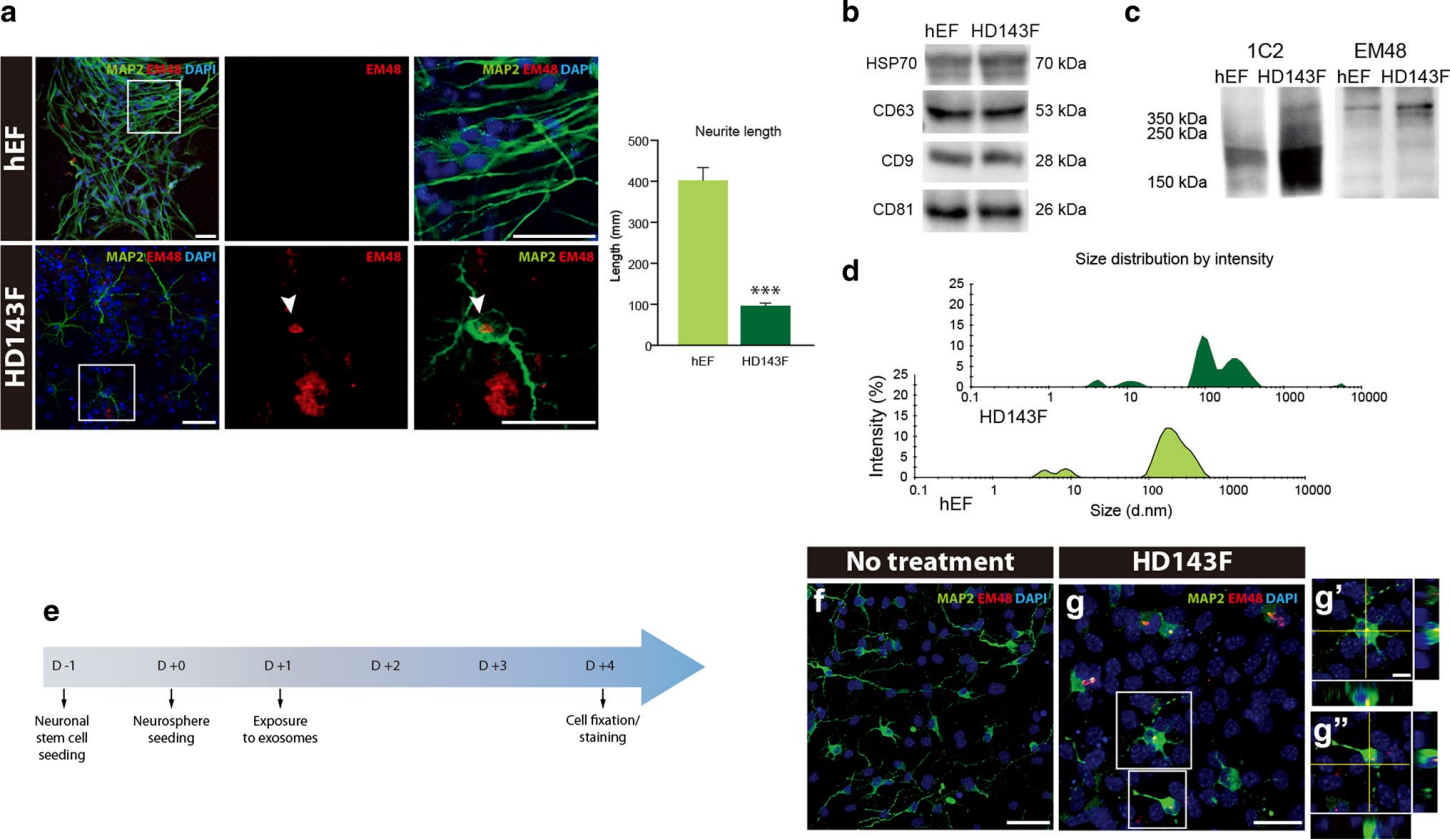
Release of mHtt by exosomes. **a** NSCs labeled with MAP2 (*green*) displayed mHtt aggregates (EM48^+^; *red*) when co-cultured with HD143F but not with hEF. Exposure of NSCs to HD143F further leads to significant morphological changes, i.e., reduction in the neurite length of NSCs (*n* = 15). Values are expressed as mean ± SEM. Statistical analyses were performed using the Student’s *t* test. ***p < 0.001 compared to hEF group. **b**, **c** Release of exosomes by fibroblasts, as confirmed by the expression of HSP70, CD63, CD9 and CD81 (**b**). mHtt labeled with either 1C2 or EM48 (**c**) is found within extracellular vesicles. **d** Analyses of the exosome-enriched fraction using a nano-sizer confirming the size of the extracted exosomes. **e** Timeline of in vitro experimentation involving co-culture of exosomes and NSCs. **f**, **g** NSCs labeled with MAP2 (*green*) and EM48 (*red*) following exposure to vehicle (**f**) and exosomes derived from HD fibroblasts (**g**, **g′**, **g″**), where images in **g** depict neuronal cells which have internalized exosomes carrying mHtt 4 days into the culture. *Scale bars* **a** = **50** µm; **f** = 50 µm; **g** = 30 µm; **g′**, **g″** = 10 µm. *D* day; *DARPP-32* dopamine- and cAMP-regulated phosphoprotein, *Mr* 32 kDa: *DAPI* 4′,6-diamidino-2-phenylindole; *GFP* green fluorescence protein; *HD143F*: fibroblasts derived from an HD patient with 143 CAG repeats; *hEF* human embryonic fibroblasts; *MAP2* microtubule associated protein 2; *mHtt* mutant huntingtin; *NSCs* neural stem cells

The identification of mHtt within host neurons suggests that mHtt can be secreted and taken up by neighbouring cells. Co-culture of neurons derived from murine NSCs with HD143F demonstrated that mHtt aggregates from diseased cells have the ability to infect and detrimentally affect normal neurons (Fig. 5a, bottom panels), with observable impacts on the morphology of exposed cells which display, in part, shorter neurites (Fig. 5a; *p* < 0.001). In contrast, when neurons are co-cultured with normal fibroblasts, neuronal differentiation is typical and no overt morphological changes are observed (Fig. 5a, top panels).

### Exosomes act as cargos in mHtt release

To determine the potential route by which mHtt is transferred to recipient cells, we investigated the release of extracellular vesicles from fibroblasts and found that, indeed, extracellular exosomes are detectable in the culture media (Fig. 5b). The release of exosomes was confirmed by immunoblots performed with different antibodies (HSP70, CD81, CD63, CD9 and CD81), as well as size analysis using Zetasizer Nano ZS (Fig. 5d), providing the first evidence that mHtt is found within extracellular vesicles (Fig. 5c). We further confirmed these results using the human neuroblastoma cell line SH-SY5Y overexpressing the exon 1 of Htt carrying 19 or 103 CAG fused to GFP or GFP alone (Supplemental Fig. 4a). The expression of mHtt was detected not only intracellularly but also in the extracellular particles identified using the anti-CD63 antibody (Supplemental Fig. 4c). The release of exosomes was further validated with Zetasizer Nano ZS (Supplemental Fig. 4d). The expression of the plasmids did not affect cell viability as demonstrated by the LDH assay (Supplemental Fig. 4b), confirming that the presence of mHtt in the extracellular media is not due to cell death.

To assess whether secreted mHtt is able to propagate from the extracellular milieu to recipient cells, we exposed SH-SY5Y cells to the conditioned media of HEK cells overexpressing GFP, GFP-mHtt-Q19 or GFP-mHtt-Q103. After 5 days of incubation, we analyzed the SH-SY5Y cells total protein fraction by western blot. The results revealed the presence of exogenous mHtt protein (Q19 and Q103) in recipient cells (Supplemental Fig. 4e). Using an anti-GFP antibody, we strictly detected mHtt, as no signal was observed for GFP alone. This suggests that propagation from the extracellular milieu to the recipient cells is specific to mHtt protein (Supplemental Fig. 4e).

To further confirm that exosomal transport could be responsible for the propagation of mHtt, neurons derived from murine NSC were exposed to HD143F derived exosomes for 4 days in culture (Fig. 5e). Differentiated cells labeled positively for MAP2 that were exposed to vesicles enriched of mHtt depicted the presence of intracellular EM48^+^ mHtt aggregates (Fig. 5g, g′, g″), while aggregates were not observed in non-treated cells that were devoid of aggregates (Fig. 5f). This finding demonstrates that cultured cells can internalize exosomes containing pathological proteins.

**Fig. 6:**
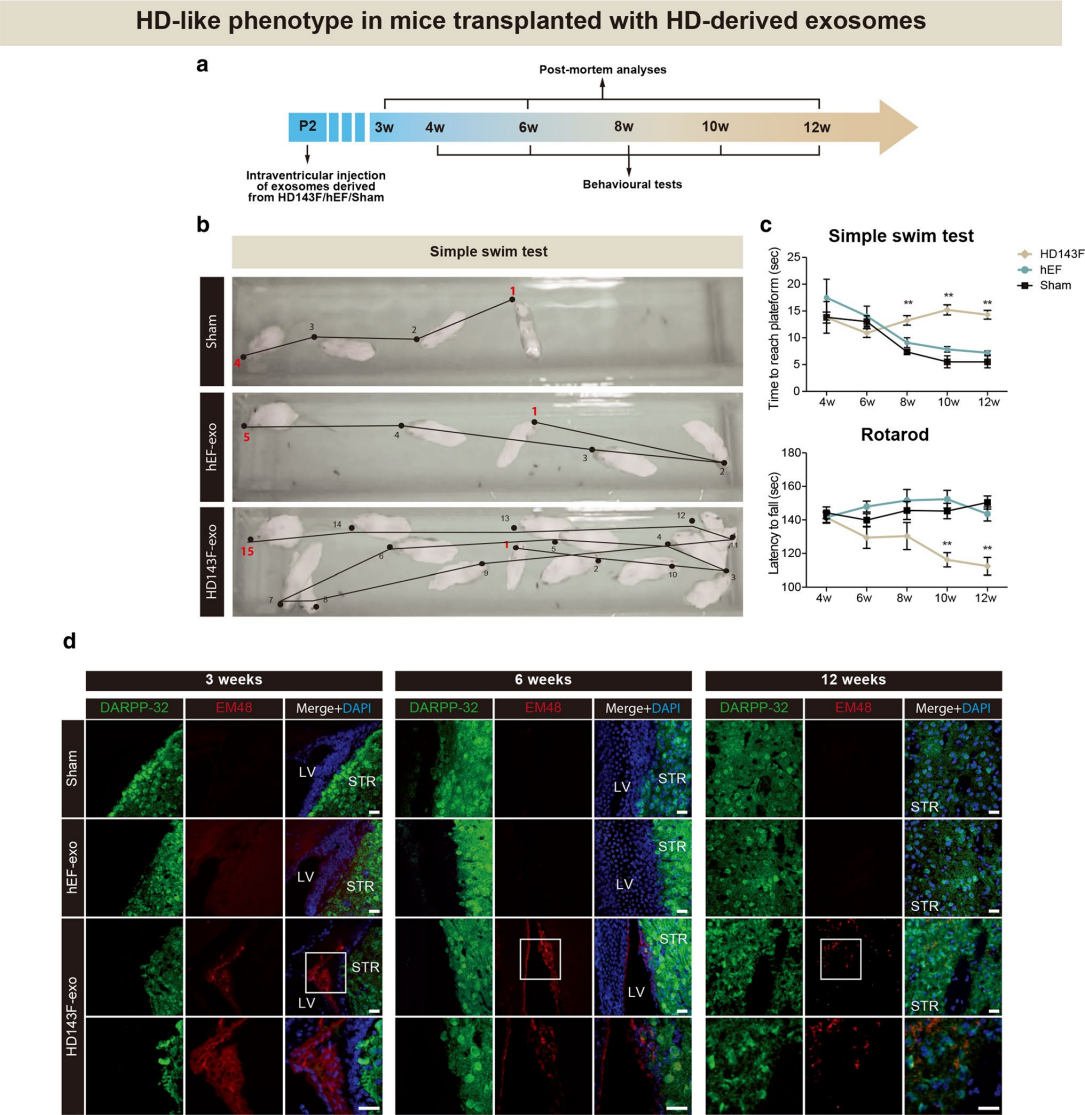
Development of HD-associated behavioural phenotypes following intraventricular injection of exosomes isolated from HD patient-derived fibroblasts and evidence of mHtt propagation within the brain. **a** Timeline of in vivo experimentation using exosomes isolated from HD143F. **b** Representative images of the simple swim test. **c** Plotting of the time to reach the platform as well as the latency to fall, as measured by the rotarod test, revealed cognitive and motor-related deficits in mice transplanted with exosomes carrying mHtt in comparison to mice injected with either exosomes derived from normal human embryonic fibroblasts or in sham animals. **d** Triple immunoflurorescent staining was further carried out for DARPP-32, EM48 and DAPI and revealed the propagation of mHtt from implanted exosomes isolated from HD patient-derived fibroblasts into the host striatum and striatal cells as early as 3 weeks post-implantation. Values are expressed as mean ± SEM. Statistical analyses were performed using the Student’s *t* test. *p < 0.05 and **p < 0.005 compared to hEF group. Numbers of mice used are as follows: hEF-exosome, *n* = 11; HD143F-exosome, *n* = 11; Sham, *n* = 9. *Scale bar* **d** = 20 µm. *DARPP-32* dopamine- and cAMP-regulated phosphoprotein, Mr 32 kDa; *DAPI* 4′,6-diamidino-2-phenylindole; *HD143F-exo* exosomes isolated from fibroblasts derived from an HD patient with 143 CAG repeats; *hEF-exo* exosomes isolated from human embryonic fibroblasts; *LV* lateral ventricle; *P2* post-natal day 2; *STR* striatum; *w* weeks

Finally, to test whether exosomes are relevant in the transport and dissemination mHtt pathology in vivo, newborn wilt-type animals were implanted with exosomes derived from HD143F, similar to what had been done for all the other experiments (Fig. 6a). As observed with the injections of fibroblasts and iPSCs, the implantation of exosomes derived from HD143F into the ventricle of neonatal mice triggered the manifestation of an HD phenotype. As early as 8 weeks post-implantation, mice injected with exosomes carrying the mHtt gene product began to show impairments in the simple swim test, which persisted to 12 weeks post-implantation, the time point at which we ceased to test the animals (Fig. 6b, c). This was accompanied by a precipitated latency to fall, as measured by the rotarod test, which was noticeable at 10 weeks post-implantation. Neither sham animals nor mice injected with exosomes derived from normal fibroblasts (hEF) showed any impairments. In the HD143F exosome-injected animals, EM48^+^ mHtt staining was detected in the striatum as early as 3 weeks post-implantation and co-localized with DARPP-32^+^ cells of the host (Fig. 6d).

## Discussion

Our manuscript provides the first evidence for (1) the propagation of the full-length mHtt protein in vivo; (2) the development of a robust HD phenotype in wild-type mice, both pathologically and behaviorally, using various cells derived from a patient with HD and that (3) exosomes can cargo mHtt both in vitro and in vivo thus being responsible, at least in part, for the propagation of mHtt.

The cell-autonomous theory of genetic disorders postulates that each cell contains an aberrant gene that contributes to individual cell pathology [15]. However, we herein demonstrate that the transfer of an abnormal gene product to genetically unrelated normal tissue is also possible, and this, with pathological consequences. This suggests that there are additional mechanisms by which genetic disorders can induce disease in tissue that does not actively express the pathology. We recently reported that mHtt protein aggregates are found within fetal striatal transplants in HD patients who underwent cell replacement therapy [7]. The results we present here are in agreement with this finding and a recent report, also by us, demonstrating that iPSCs isolated from an HD patient can give rise to HD-like cellular pathology after an extended in vivo exposure into healthy neonatal brains [21].

There is now mounting evidence that pathological species of proteins can be transported trans-synaptically and generate relevant disease phenotypes [22, 44]. In vivo observations using human neurons implanted in the cortex of R6/2 mice has suggested mHtt transfer between synaptically connected neuronal elements [37]. Blocking the synaptic vesicles fusion with botolinum neurotoxins prevented the spreading of mHtt in organotypic cultures [37]. The identification of pathology remote from the injection sites supports an intercellular trans-synaptic protein transmission as do studies showing expression of human α-synuclein in rodent allografts transplanted in animals expressing human α-synuclein [2]. In the latter study, human α-synuclein co-localized with markers of endosomes and exosomes [2], which could represent the route by which this protein is transferred [2, 16]. In addition, exosomal markers have been found to be associated with amyloid plaques in Alzheimer’s disease (AD) [42] and in vitro models of Amyotrophic Lateral Sclerosis have revealed that misfolded SOD1 transmission can also occur via released exosomes [17]. The identification of mHtt aggregates within our cultured NSCs exposed to mHtt containing exosomes is in line with these studies and the development of a full-blown HD phenotype in mice injected with exosomes derived from diseased fibroblasts strongly supports the role of exosomes as vehicle of propagation of HD pathology.

In HD, evidence that mHtt can propagate from cell to cell has only recently emerged, with the vast majority of results derived from in vitro studies [9, 20, 43, 47]. More specifically, various cell types, including neuronal-like cells (COS7, HEK293T and PC12 cell lines) have been shown to take up and internalize synthetic mHtt aggregates exogenously delivered to the culture milieu [43, 47]. In these experiments, aggregates were localized within the cytoplasm and associated with proteins involved in the quality control of the cell, such as ubiquitin [43]. When the synthetic protein was tagged with a sequence mediating its import into the nuclear compartment, aggregates were translocated to the nucleus, where it leads to cell death [47]. More recently, another group developed a Biomolecular Fluorescence Complementation-based assay using non-fluorescent halves of the Venus protein tagged to mHtt. When mHtt underwent oligomerization, a fluorescent signal was detected. To evaluate whether mHtt could propagate from cell to cell, the two halves were expressed independently in two separate cell populations. The mixed population presented a significant number of fluorescent cells, which demonstrated the capacity of the protein to migrate from cell to cell but also to act as a seed for further protein aggregation [20].

In many sporadic neurodegenerative diseases, such as AD and PD, the propagation of abnormal/pathological proteins has been proposed to occur in a prion-like fashion [1, 16]. Such prion-like spread has recently been suggested to take place following the phagocytosis of mHtt aggregates by glia in a *Drosophila* model of HD, in which engulfed aggregates were granted access to the glial cytoplasm where they interacted with soluble Htt, initiating a prion-like dissemination of the pathology [36]. This suggested that phagocytosis, otherwise neuroprotective in neurodegenerative disorders, could contribute to deleterious non-cell-autonomous effects. More recently, mHtt was also shown to accumulate at the synaptic terminals of the olfactory receptor neurons in another *Drosophila* model of HD with subsequent migration and diffusion through the brain. The mutant protein was eventually internalized by other neurons, thereby leading to non-cell-autonomous degeneration. Interestingly, differential expression of mHtt in different neuronal populations leads to different patterns of propagation [3]. However, critically, none of these papers have established a direct link between protein spread and the development of a full-blown HD-like phenotype. Our results, therefore, represent a significant advancement in the possible prion-like spread of mHtt by demonstrating that pathology can be induced and propagated in the mammalian brain with the development of HD in healthy mice, and importantly, serve as a new model to further investigate how abnormal proteins can be transferred from cell to cell and lead to pathological features in vivo.

While our observations do not negate the cell-autonomous action of mHtt in HD pathogenesis, they do suggest that, even in genetic disorders of the CNS, protein spread can occur and may be important in the pathogenic propagation of disease. Our results have profound implications for a whole range of CNS diseases as well as opening up new therapeutic avenues for HD and neurodegenerative disorders characterized by similar proteinopathies.

## Electronic supplementary material

Below is the link to the electronic supplementary material.

Supplemental Figure 1. Characterization of HD143F. **(a)** Schematic representation of the *Htt* locus present in HD and control fibroblasts. The primers Htt-UTR FW and Htt intron 1-2 Rev were used to amplify exon 1 of the *Htt* gene and to confirm the presence of the elongated CAG repeat in HD143F. **(b-c)** The expression of mHtt was confirmed by **(b)** immunoblot using antibodies raised against the polyQ region (1C2) and total Htt **(c)** as well as immunofluorescent staining for mHtt (EM48; red). (**d**) Karyotypes for hEF and HD143F cells as well as (**e**) the growth curve for both cell types. Scale bar: **c** = 20 µm. Abbreviations: DAPI, 4’,6-diamidino-2-phenylindole; HD143F, fibroblasts derived from an HD patient with 143 CAG repeats; hEF, human embryonic fibroblasts; htt, huntingtin; UTR, untranslated region (TIFF 8678 kb)

Supplemental Figure 2. Characterization of HD143-iPSC. Triple immunofluorescent staining for mHtt (EM48; red), Nestin (green) and DAPI (blue) in epi-iPSC and HD143-iPSC at both the neuroal precursor stage (**a**) and neuronal cell stage (**b**). (**c**) Graph depicting impairments of neurite growth in diseased HD143-iPSC. Values are expressed as means ± S.E.M. Statistical analyses were performed using Student’s t-test. * = *p* < 0.05 to epi-iPSC group. (**d**) Karyotypes for epi-iPSC and HD143-iPSC. Scale bars **a, b** = 50 µm. Abbreviations: DAPI, 4’,6-diamidino-2-phenylindole; epi-iPSC, induced pluripotent stem cells derived from normal fibroblasts; HD143-iPSC, induced pluripotent stem cells derived from the HD143F (TIFF 21281 kb)

Supplemental Figure 3. Development of HD-associated behavioural phenotypes following intraventricular injection of HD patient-derived fibroblasts and iPSCs. **(a)** Timeline of *in vivo* experimentation using fibroblasts derived from HD patients carrying either 72 or 180 CAG repeats. **(b)** Representative images of the simple and forced swim tests. (**c**) Quantification of the time to reach the platform in the simple swim test, immobility time on the forced swim test, the forelimb grip strength as well as the latency to fall on the rotarod test, which were all revealed as HD phenotypes in mice transplanted with fibroblasts collected from HD patients (HD72F or 180F) compared to animals in receipt of control cells (hEF). Values are expressed as means ± S.E.M. Statistical analyses were performed using One-way ANOVA followed by a Tukey post-doc tests. * = *p* < 0.05 and ** = *p* < 0.005 compared to the sham group; # = *p* < 0.05 compared to the HD180F group. The number of mice used in each group is indicated in each column of the graphs. **(d)** Timeline of experimentation involving HD patient-derived iPSCs. **(e)** Recorded trajectories on the simple swim test of representative mice transplanted with iPSC from an HD patient (HD143-iPSC) or control individual (epi-iPSC) as well as images of the forced swim test. (**f**) Quantification of the time to reach the platform (simple swim test), immobility time (forced swim test), forelimb grip strength as well as the latency to fall (rotarod) further confirmed an HD-like phenotype in animals transplanted with HD patient-derived iPSCs. Values are expressed as means ± S.E.M. Statistical analyses were performed using Student’s t-test. * = *p* < 0.05 compared to HD143-iPSC groups. The number of mice used in each group is indicated in each column of the graphs. Abbreviations: epi-iPSC, induced pluripotent stem cells derived from normal fibroblasts; HD72F, fibroblasts derived from an HD patient with 72 CAG repeats; HD180F, fibroblasts derived from an HD patient with 180 CAG repeats; HD143-iPSC, induced pluripotent stem cells derived from the HD143F; hEF, human embryonic fibroblasts; P2: post-natal day 2; w, weeks (TIFF 20982 kb)

Supplemental Figure 4. Exosomes act as cargos in mHtt release. **(a)** Expression of plasmids carrying either GFP or exon 1 with 19 or 103 CAG repeats tagged with GFP in SH-SY5Y cells. **(b)** LDH assay showing comparable viability of SH-SY5Y cells after transfection. **(c)** Expression of Htt exon 1 with 19 or 103 CAG repeats tagged with GFP in SH-SY5Y cells and released exosomes (n=3 repetitions). **(d**) Analyses of the exosome-enriched fraction using a nanosizer confirming the size of extracted exosomes in the extracellular media. (**e**) Uptake of mHtt proteins from the extracellular media. SH-SY5Y cells were incubated in conditioned media from HEK cells overexpression mHtt (Q19 and Q103) fused to GFP. Total protein fraction was analyzed by western blot using anti-mHtt (EM48) and anti-GFP antibodies Statistical analyses were performed using One-way ANOVA. ** *p* < 0.005. Western blot bands are from the same experiment and were cropped from the same membrane (TIFF 4976 kb)

Supplemental Video 1. Clasping test in mice injected with HD143F or hEF at 30 weeks post-injection (MP4 3179 kb)

Supplemental Video 2. Simple swim test in mice injected with HD143F or hEF at 30 weeks post-injection (MP4 8913 kb)

Supplemental Video 3. Simple swim test in mice injected with HD143-iPSC or epi-iPSC at 36 weeks post-injection (MP4 16572 kb)

Supplemental Video 4. Forced swim test in mice injected with HD143-iPSC or epi-iPSC at 36 weeks post-injection (MP4 18897 kb)
