# Supplementary figures and images for: Human-to-mouse prion-like propagation of mutant huntingtin protein
Source: Acta Neuropathol. 2016 May 24;132(4):577–92. doi: 10.1007/s00401-016-1582-9 (PMC5023734; doi:10.1007/s00401-016-1582-9)

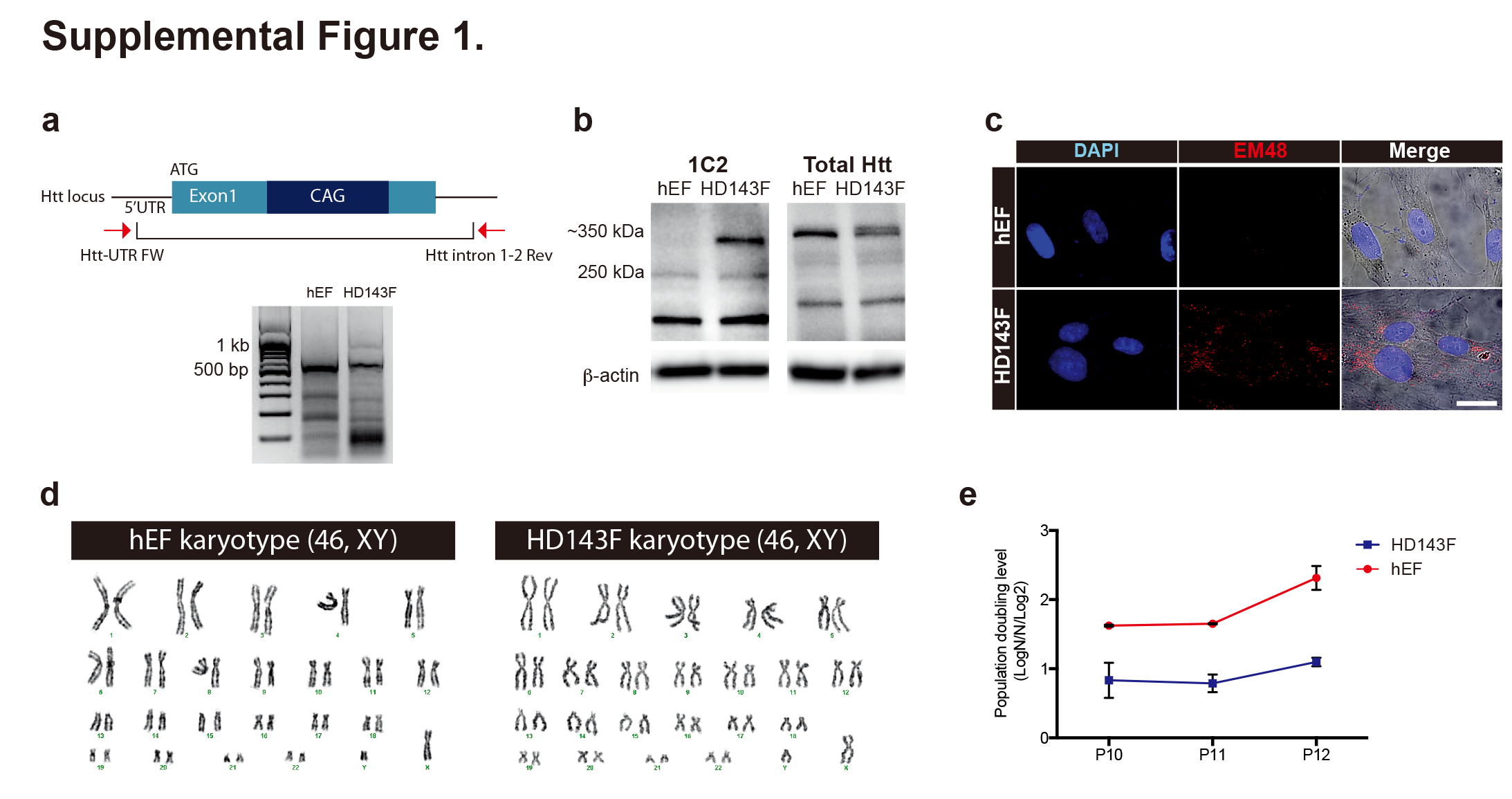

Supplement: Supplementary file 1 — Supplemental Figure 1. Characterization of HD143F. (a) Schematic representation of the Htt locus present in HD and control fibroblasts. The primers Htt-UTR FW and Htt intron 1-2 Rev were used to amplify exon 1 of the Htt gene and to confirm the presence of the elongated CAG repeat in HD143F. (b-c) The expression of mHtt was confirmed by (b) immunoblot using antibodies raised against the polyQ region (1C2) and total Htt (c) as well as immunofluorescent staining for mHtt (EM48; red). (d) Karyotypes for hEF and HD143F cells as well as (e) the growth curve for both cell types. Scale bar: c = 20 µm. Abbreviations: DAPI, 4’,6-diamidino-2-phenylindole; HD143F, fibroblasts derived from an HD patient with 143 CAG repeats; hEF, human embryonic fibroblasts; htt, huntingtin; UTR, untranslated region (TIFF 8678 kb) [file 401_2016_1582_MOESM1_ESM.tif]

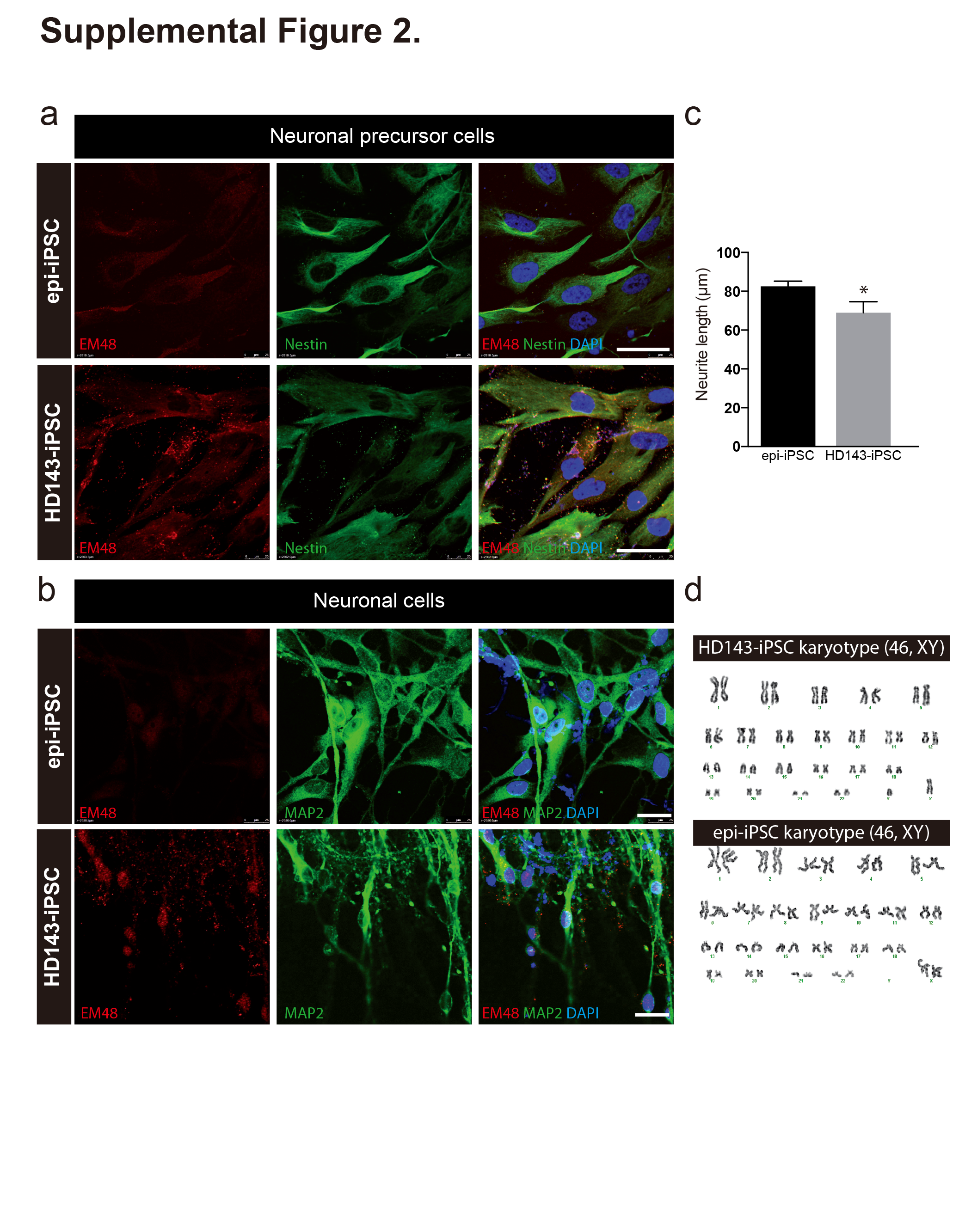

Supplement: Supplementary file 2 — Supplemental Figure 2. Characterization of HD143-iPSC. Triple immunofluorescent staining for mHtt (EM48; red), Nestin (green) and DAPI (blue) in epi-iPSC and HD143-iPSC at both the neuroal precursor stage (a) and neuronal cell stage (b). (c) Graph depicting impairments of neurite growth in diseased HD143-iPSC. Values are expressed as means ± S.E.M. Statistical analyses were performed using Student’s t-test. * = p < 0.05 to epi-iPSC group. (d) Karyotypes for epi-iPSC and HD143-iPSC. Scale bars a, b = 50 µm. Abbreviations: DAPI, 4’,6-diamidino-2-phenylindole; epi-iPSC, induced pluripotent stem cells derived from normal fibroblasts; HD143-iPSC, induced pluripotent stem cells derived from the HD143F (TIFF 21281 kb) [file 401_2016_1582_MOESM2_ESM.tif]

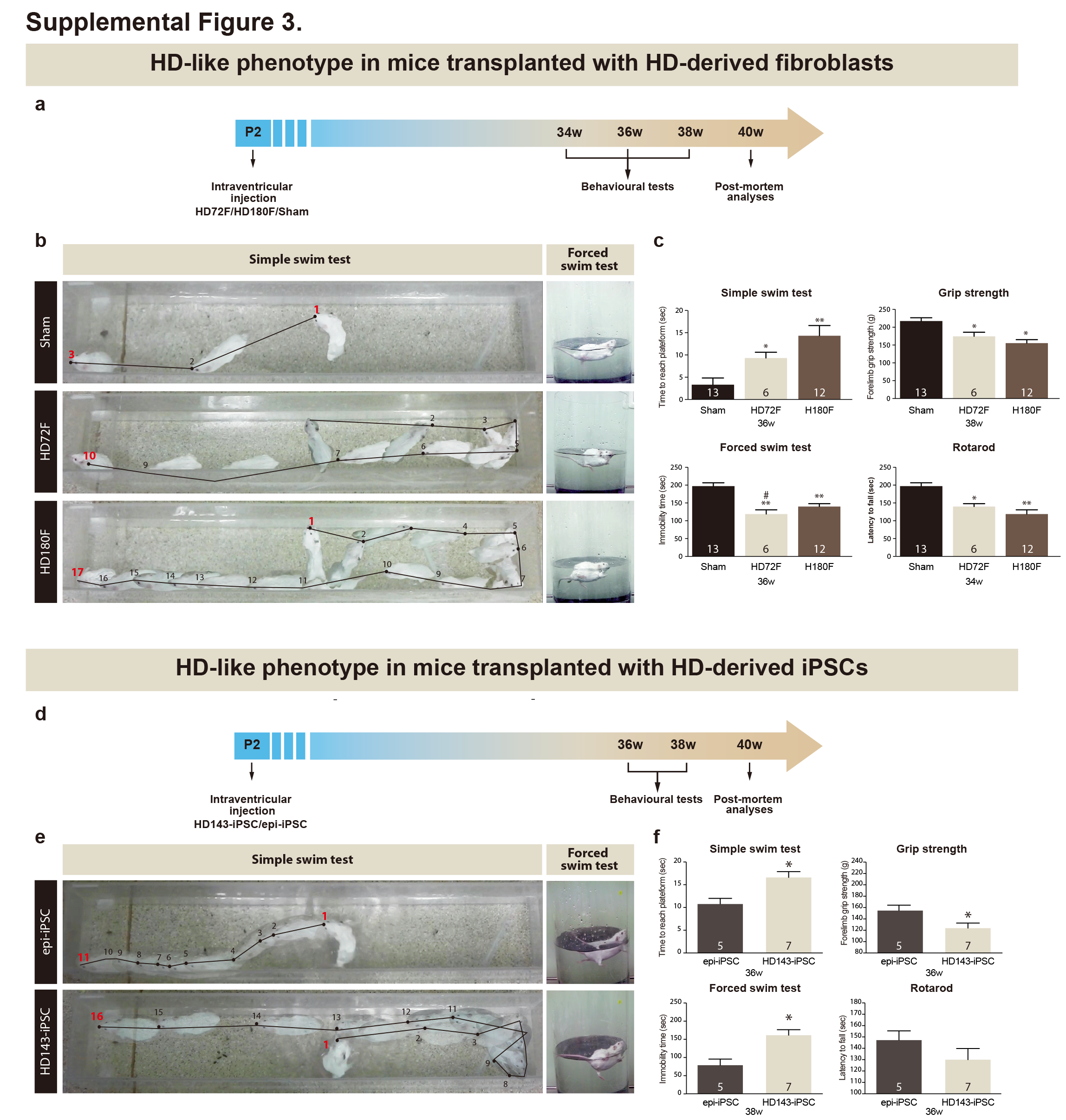

Supplement: Supplementary file 3 — Supplemental Figure 3. Development of HD-associated behavioural phenotypes following intraventricular injection of HD patient-derived fibroblasts and iPSCs. (a) Timeline of in vivo experimentation using fibroblasts derived from HD patients carrying either 72 or 180 CAG repeats. (b) Representative images of the simple and forced swim tests. (c) Quantification of the time to reach the platform in the simple swim test, immobility time on the forced swim test, the forelimb grip strength as well as the latency to fall on the rotarod test, which were all revealed as HD phenotypes in mice transplanted with fibroblasts collected from HD patients (HD72F or 180F) compared to animals in receipt of control cells (hEF). Values are expressed as means ± S.E.M. Statistical analyses were performed using One-way ANOVA followed by a Tukey post-doc tests. * = p < 0.05 and ** = p < 0.005 compared to the sham group; # = p < 0.05 compared to the HD180F group. The number of mice used in each group is indicated in each column of the graphs. (d) Timeline of experimentation involving HD patient-derived iPSCs. (e) Recorded trajectories on the simple swim test of representative mice transplanted with iPSC from an HD patient (HD143-iPSC) or control individual (epi-iPSC) as well as images of the forced swim test. (f) Quantification of the time to reach the platform (simple swim test), immobility time (forced swim test), forelimb grip strength as well as the latency to fall (rotarod) further confirmed an HD-like phenotype in animals transplanted with HD patient-derived iPSCs. Values are expressed as means ± S.E.M. Statistical analyses were performed using Student’s t-test. * = p < 0.05 compared to HD143-iPSC groups. The number of mice used in each group is indicated in each column of the graphs. Abbreviations: epi-iPSC, induced pluripotent stem cells derived from normal fibroblasts; HD72F, fibroblasts derived from an HD patient with 72 CAG repeats; HD180F, fibroblasts derived from an HD patient wi [file 401_2016_1582_MOESM3_ESM.tif]

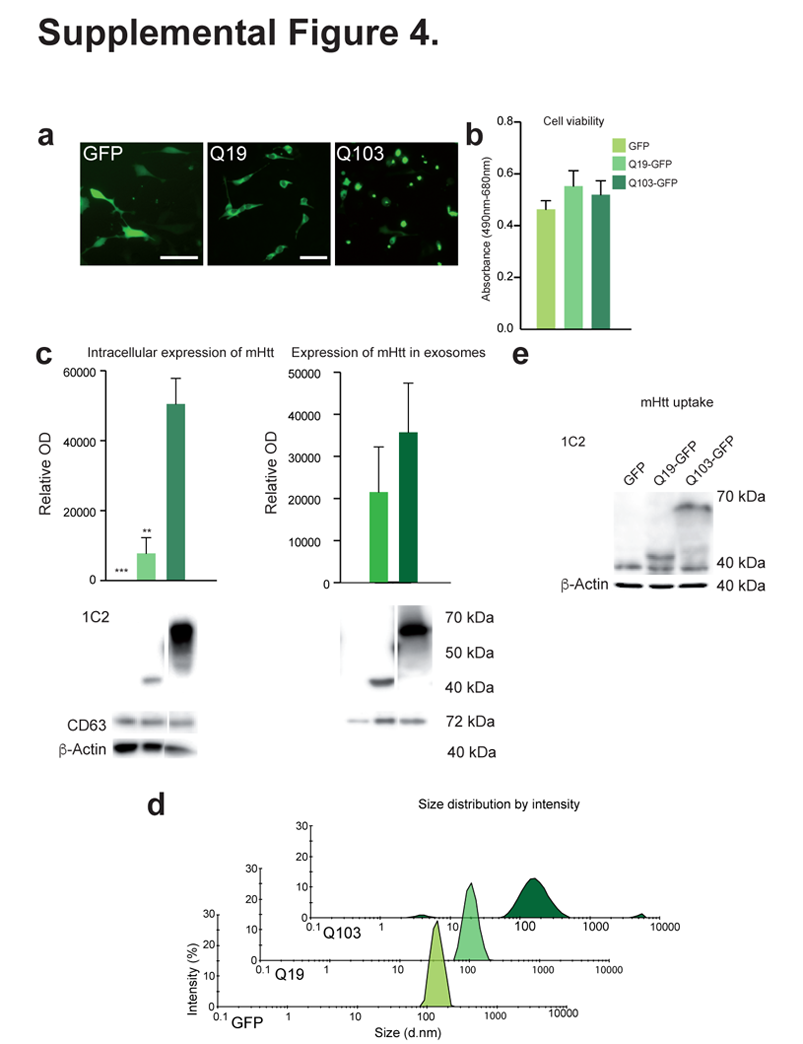

Supplement: Supplementary file 4 — Supplemental Figure 4. Exosomes act as cargos in mHtt release. (a) Expression of plasmids carrying either GFP or exon 1 with 19 or 103 CAG repeats tagged with GFP in SH-SY5Y cells. (b) LDH assay showing comparable viability of SH-SY5Y cells after transfection. (c) Expression of Htt exon 1 with 19 or 103 CAG repeats tagged with GFP in SH-SY5Y cells and released exosomes (n=3 repetitions). (d) Analyses of the exosome-enriched fraction using a nanosizer confirming the size of extracted exosomes in the extracellular media. (e) Uptake of mHtt proteins from the extracellular media. SH-SY5Y cells were incubated in conditioned media from HEK cells overexpression mHtt (Q19 and Q103) fused to GFP. Total protein fraction was analyzed by western blot using anti-mHtt (EM48) and anti-GFP antibodies Statistical analyses were performed using One-way ANOVA. ** p < 0.005. Western blot bands are from the same experiment and were cropped from the same membrane (TIFF 4976 kb) [file 401_2016_1582_MOESM4_ESM.tif]
